# Evidence-based self-medication: development and evaluation of a professional newsletter concept for community pharmacies

**DOI:** 10.1007/s11096-020-01100-6

**Published:** 2020-07-30

**Authors:** Katharina Moritz, Jasmin Mina Seiberth, Susanne Schiek, Thilo Bertsche

**Affiliations:** 1 Department of Clinical Pharmacy, Institute of Pharmacy, Faculty of Medicine, Leipzig University, Brüderstr. 32, 04103 Leipzig, Germany; 2 Drug Safety Center, University Hospital Leipzig and Leipzig University, Brüderstr. 32, 04103 Leipzig, Germany

**Keywords:** Continuing education, Evidence-based pharmacy practice, Germany, Pharmacies, Self-medication, Surveys and questionnaires

## Abstract

*Background *Providing evidence-based care is recognized as a key competence for all healthcare professionals. In order to support community pharmacists in evidence-based self-medication counseling, the umbrella organization of German pharmacists initiated the development of a nationwide concept. The key element of the concept was a professional newsletter that should help pharmacists incorporate research findings into their daily counseling practice. *Objective* To develop, implement and evaluate the professional newsletter concept. *Setting* German community pharmacies. *Method* Clinical pharmacists from a German university compiled and synthesized clinical trial data in a professional newsletter that would supply community pharmacists with evidence-based information on common over-the-counter medicines as well as instructions for searching and appraising scientific literature. The electronic newsletter was offered to interested community pharmacists free of charge, once or twice a month, after they signed up for a subscription. About one year after the publication of the first newsletter issue, the subscribers were invited to take part in an anonymous cross-sectional online survey. In all, 21 newsletter issues were published through the end of the survey period. *Main outcome measure* Perceived value of the professional newsletter with 10 predefined objectives. *Results* A total of 1975 persons subscribed to the professional newsletter. Of those, 150 persons working in community pharmacies completed the survey. Most of them perceived the synthesized information as ‘useful’ (81–95%). They attributed positive changes in knowledge (89%), skills (87–91%), awareness (85%), and motivation (67–77%) to the newsletter. However, almost half of them (43%) found it difficult to incorporate reading the newsletter in their everyday working life. Free-text feedback suggested that further modifications should be considered to facilitate a better integration of the newsletter into everyday pharmacy practice. *Conclusion* A nationwide provided professional newsletter can play a vital part in supporting pharmacists in evidence-based self-medication counseling. However, the practicability of such a newsletter needs to be further improved and the newsletter should be accompanied by additional measures.

**Electronic supplementary material:**

The online version of this article (10.1007/s11096-020-01100-6) contains supplementary material, which is available to authorized users.

## Impacts of practice

- A professional newsletter is a possible and widely accepted resource by community pharmacists for evidence-based information on OTC medicines.
- Information resources for evidence-based self-medication counseling should both summarize clinical trial data and give instructions for the critical appraisal of scientific literature.
- Professional newsletter concepts should facilitate pharmacists to deal with evidence-based information in their daily practice and to counsel patients at the counter, using the provided information.

## Introduction

Implementing an evidence-based approach is a matter of course in physicians’ everyday prescribing routine [1]. Evidence from clinical trials should form the foundation of all healthcare professionals’ advice. At the same time, their own clinical experience and patient preferences should be taken into account [2]. Pharmaceutical associations worldwide have recommended that pharmacy practice should follow the same principles [3, 4]. To support implementation of evidence-based principles in the community pharmacy in Germany, a perspective paper was developed in a bottom-up manner by more than 4000 pharmacists [5]. This is particularly important, because pharmacists have not yet consistently implemented an evidence-based approach to their counseling practice [6–12]. They face difficulties in incorporating research findings into practice [6]. Despite that pharmacists are obliged and encouraged to pursue continuing education [13, 14], several studies have demonstrated a lack of pharmacists’ knowledge and skills as major obstacles to the practice of evidence-based care [7, 8, 15]. In addition, due to lack of time in routine care, pharmacists have limited motivation to read original publications of clinical trials [6, 16, 17]. Hence, tailored instruments are necessary to provide scientific evidence to them in their everyday working life.

Community pharmacists often are the primary healthcare professional contact for patients seeking advice, especially for self-medication [18], resulting in a considerable responsibility. However, applicable evidence-based resources for self-medication counseling have been lacking [7, 15]. Therefore, at the request of their members [19], the Federal Union of German Associations of Pharmacists (ABDA), the umbrella organization of German pharmacists, initiated the development of a nationwide concept. This concept was to be based on the core principles of evidence-based practice (EBP: i.e., ask, acquire, appraise, apply, and assess) [20]. Producing an EBP professional newsletter was anticipated to be an appropriate core strategy by the ABDA and clinical pharmacists from Leipzig University. This was a pragmatic approach, since pharmacists prefer to read specialized literature that allows them to educate themselves independently rather than through fixed scheduled training [6]. Besides, providing a professional newsletter would serve as continuing education for many pharmacists with relatively small personnel and logistic effort [21]. It was hypothesized that the provision of evidence-based information with instructions for searching and appraising scientific literature would support and motivate pharmacists to use evidence-based principles in their counseling practice. Professional newsletter concepts that focus on the presentation of trial data are already established in the context of continuing medical education. Results from other studies indicated that physicians appreciated the provision of professional newsletters with summarized and critically appraised clinical trial data [22] and that such newsletters improved their prescribing behavior [23]. Hence, we supposed that community pharmacists also could be supported in EBP by similar information resources.

## Aim of the study

We aimed to develop, implement and evaluate a nationwide EBP professional newsletter to support pharmacists in evidence-based self-medication counseling. The evaluation would examine the subscribers' assessment of the information presentation in the newsletter, the self-perceived influence of the newsletter on their skills, knowledge, awareness and motivation regarding evidence-based counseling, as well as barriers to the newsletter’s integration in everyday practice.

## Ethics approval

According to the regulations of the ethics committee of the Leipzig Medical Faculty, an ethics approval is necessary only if epidemiological research includes personally identifiable information. This was not the case in our anonymous online questionnaire survey of newsletter subscribers. Participation in the study was voluntary. Consent to participate was assumed by the completion of the survey.

## Method

### Development of the professional newsletter concept

**Fig. 1:**
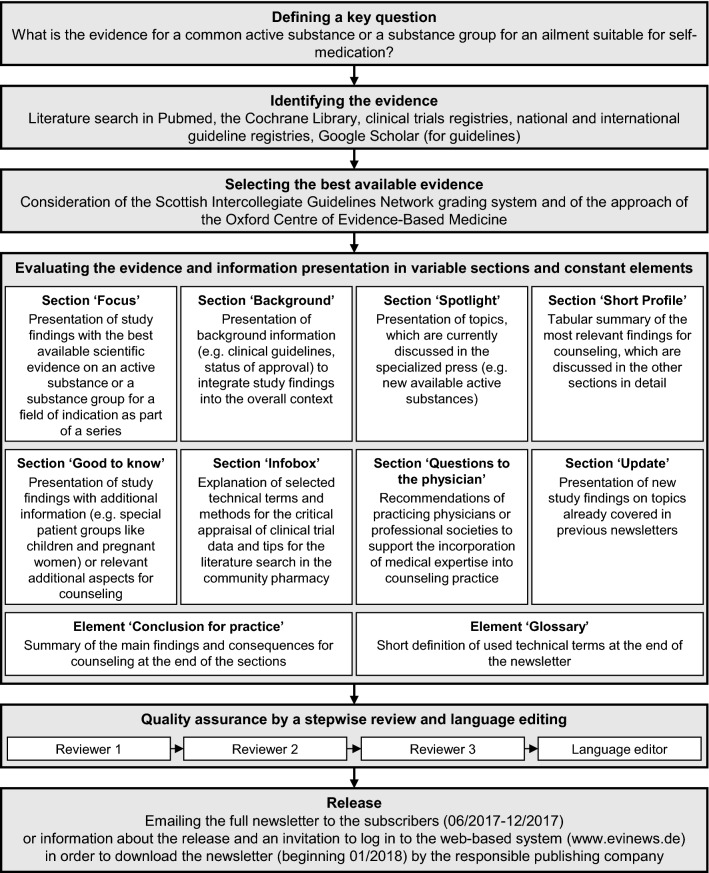
Steps for the creation of the newsletter issues. The four writers and reviewers were employed by a German university (Leipzig University, Department of Clinical Pharmacy). The language editor was employed by the responsible publishing company (AVOXA—Media Group German Pharmacist GmbH, Eschborn, Germany)

The professional newsletter (named ‘EVInews’) provided evidence-based information on common over-the-counter (OTC) medicines as well as instructions for searching and appraising scientific literature. In the newsletter, the information was presented in variable sections (these occurred with varying frequency in the newsletter) and constant elements (these occurred in each newsletter). In total, five pharmacists were involved in draft writing, draft reviewing and language editing (Fig. 1). The four writers and reviewers had vast experience in counseling patients for self-medication. To deepen their knowledge further in EBP, they attended several training sessions (e.g., for postgraduate specialization in the area of drug information). The professional language editor had more than 20 years of experience as managing and copy editor. In order to assure an independent report, the writers and reviewers from the university received no remuneration from pharmaceutical companies. The professional newsletter was further free of any commercial advertisement (e.g., by pharmaceutical companies).

The professional newsletter was promoted repeatedly by local Chambers of Pharmacists and by the ABDA in their professional pharmaceutical magazines. Its first issue was published in print as a supplement in a professional pharmaceutical magazine on May 23, 2017 in order to attract the attention of community pharmacists and to encourage them to subscribe to the professional newsletter online, free of charge. Subsequent newsletter issues were published electronically. Initially, the professional newsletter was published twice a month. In view of the personnel effort involved in producing a newsletter issue, this frequency of publication was decreased to once a month beginning December 2017. Until the end of the survey period, in total, 21 newsletter issues addressing various topics (e.g., medications for pain and the common cold, effect measures in clinical trials such as the number needed to treat and odds ratio) were published. The median page count of the newsletter issues was 9.6 pages. Online Resource 1 provides information about the topics and page count of each newsletter issue.

### Evaluation survey

#### Participants and setting

Eligible survey participants were newsletter subscribers involved in patient counseling in German community pharmacies. Subscribers who did not state they worked in a community pharmacy during the survey were excluded from the final analyses. The precise number of subscribers working in community pharmacies is not available since this information was not mandatory for the newsletter subscription. In the survey period, 1975 persons subscribed to the professional newsletter. On the basis of response rates in other online surveys among community pharmacists in Germany [24, 25], we aimed to reach a sample size of at least 8–10% of all subscribers.

#### Development of the survey

Four pharmacists developed the survey. To ensure comprehensibility and feasibility, the survey was pretested stepwise with four further pharmacists not involved in the development of the survey. These pharmacists were instructed to comment on questions that they assessed as unclearly articulated, and on response options that they assessed as irrelevant or incomprehensible. They also were asked to comment on technical problems that arose during the completion of the survey. Modifications, such as revising the wording, were made based on the received feedback. The results of the pretests were not included in the final data assessment.

The final survey (Online Resource 2), included questions on the following: (1) participants’ characteristics (socio-demographic data and usage behavior); (2) assessment of the information presentation in the professional newsletter; (3) perceived value of the professional newsletter with 10 predefined objectives about its influence on the subscribers’ knowledge, skills, awareness and motivation as well as the practicability of reading (main outcome); and (4) barriers to the professional newsletter’s integration in everyday practice and suggestions for modifications to the concept. Three-point and 6-point Likert-scales, dichotomous answer options as well as free-text options were used to obtain the participants’ opinions and usage of the newsletter.

#### Data collection

Data collection took place anonymously in a cross-sectional format from March 13 to July 31, 2018. From March to July 2018, each of the five released newsletter issues contained an invitation to participate in the survey. Additionally, six reminders were sent to all subscribers via email by the responsible publishing company and a note was placed on www.evinews.de, which appeared after the subscriber logged in to the web-based system. The survey was available at www.soscisurvey.de [26].

#### Data analysis

Spearman's rank-order correlation was conducted to explore the relationship between achieving the 10 objectives on a 6-point Likert-scale and the number of newsletter issues read. For the purpose of this analysis, the midpoint of the stated value range for the number of issues read was used. Missing data were deleted pairwise and Spearman’s rho was calculated. Calculated Spearman’s rho values of *ρ* were classified according to Cohen et al. (|ρ|= 0.100–0.299 small, |ρ|= 0.300–0.499 medium, |ρ|≥ 0.500 large) [27]. Stated reasons in the free-text boxes for non-achievement of the 10 objectives were thematically assigned to four predefined categories: personal reasons, patient-related reasons, underlying conditions, and newsletter-related reasons. In order to identify possible improvement of the newsletter’s practicability, two researchers screened and discussed the responses in the category ‘newsletter-related reasons’. They classified the individual responses in five subcategories of suggestions: shortening, additional short text version, simplifying the content, intensified graphical editing and incorporation in pharmacy software. For each subcategory, they quoted examples to illustrate the suggestions. The data analysis was conducted using IBM SPSS Statistics Version 25.0. The threshold for statistical significance was set at *P* < 0.05.

## Results

### Participants’ characteristics

The survey was sent to all 1975 newsletter subscribers. In total, 179 participants completed it, corresponding to a sample of 9% of all subscribers. From those, 29 participants were excluded from the final analysis, as they did not state they worked in a community pharmacy. At the end, 150 participants with a median work experience of 20 years in community pharmacies were included in the final analysis. Taking into account their weekly working time in the pharmacy, 36% and 53% worked ‘always’ and ‘frequently’ in counter sales, respectively (Table 1).

**Table 1:** Characteristics of survey participants from community pharmacies (n [total] = 150)

Characteristics	Values	
*Socio-demographic data*		
Median age (years [Q25/Q75])	46	38/55
Not specified (n [%])	1	1%
Gender female (n [%])	81	54%
Profession Pharmacist (n [%])	142	95%
Owner of a community pharmacy (n [%])	57	38%
Median work experience in the community pharmacy (years [Q25/Q75])	20	11/28
Frequency of activity in counter sales		
Always (n [%])	54	36%
Frequently (n [%])	79	53%
Sometimes (n [%])	15	10%
Seldom (n [%])	1	1%
Never (n [%])	1	1%
*Usage behavior*		
Median duration of newsletter subscription (months [Q25/Q75])	10	7/12
Not specified (n [%])	1	1%
Number of read newsletter issues		
More than 15 (n [%])	16	11%
11 to 15 (n [%])	20	13%
6 to 10 (n [%])	59	39%
1 to 5 (n [%])	53	35%
0 (n [%])	2	1%
Time frame of reading		
Rather in working hours (n [%])	56	37%
Rather in leisure time (n [%])	61	41%
Both apply equally (n [%])	31	21%
Not asked as no newsletter issue was read (n [%])	2	1%
Mode of reading (elaborateness)		
Rather skimming through it (n [%])	44	29%
Rather working through it (n [%])	14	9%
Both apply equally (n [%])	90	60%
Not asked as no newsletter issue was read (n [%])	2	1%
Forms of usage (multiple forms possible)		
To compile counseling recommendations (n [%])	69	46%
As a general work of reference (n [%])	56	37%
As a basis for further searches (n [%])	53	35%
To exchange our views about the newsletter in the pharmacy team (n [%])	49	33%
Other (n [%])	8	5%

The rounding of values may result in total amounts deviating from 100%

*Q25* first quartile, *Q75* third quartile

### Assessment of the information presentation

**Fig. 2:**
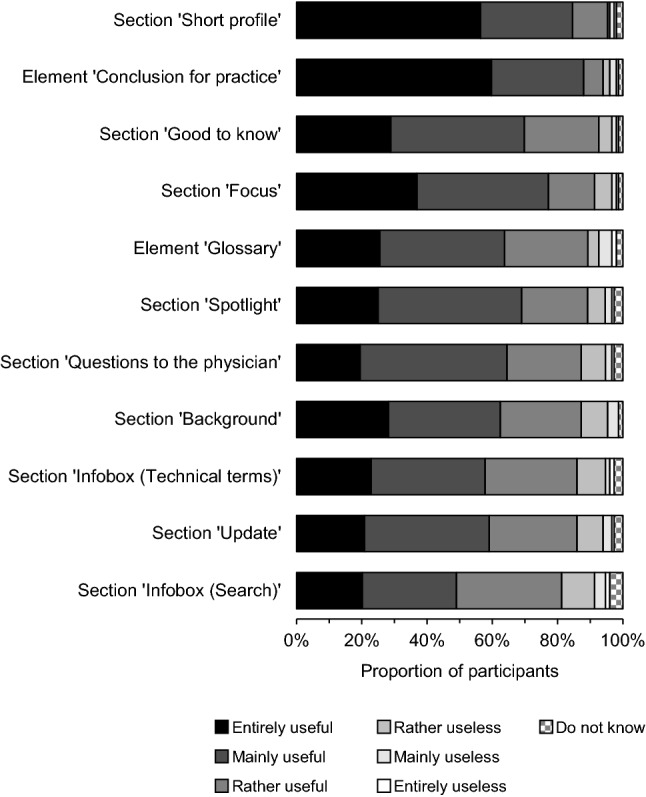
Participants’ ratings of the usefulness of the sections and elements in the newsletter. The sections and elements of the newsletter are arranged in descending order according to the proportion of participants assessing them as ‘useful’. Data on the item ‘Spotlight’ were missing for two participants. Data on each other item were missing for one participant (n [total] = 150)

Figure 2 summarizes the survey results relating to the participants’ ratings of the usefulness of the sections and elements in the professional newsletter. The presentation of information in the different sections and elements was perceived as ‘(entirely/mainly/rather) useful’ by 81–95% of the participants.

### Perceived value of the professional newsletter

**Fig. 3:**
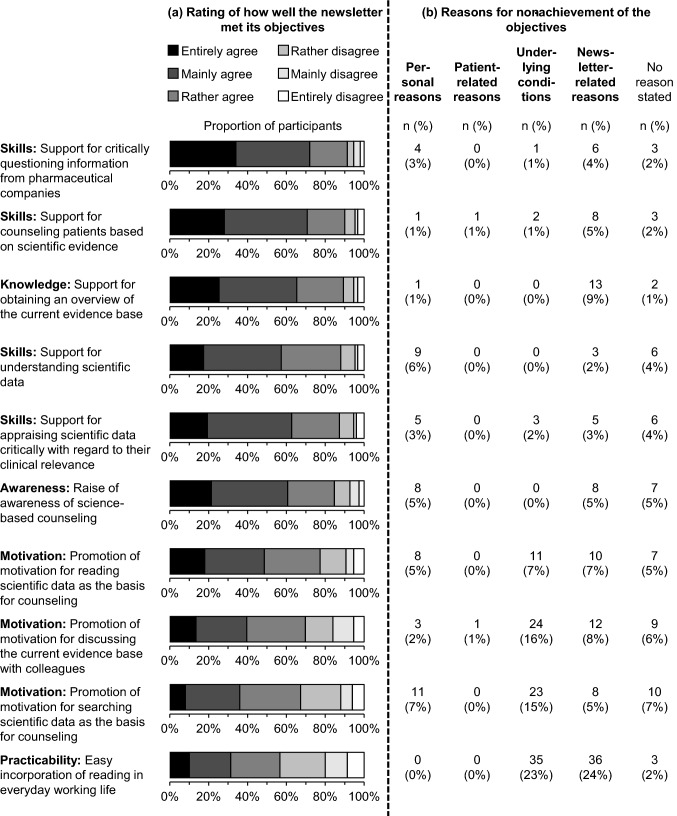
**a** Participants’ ratings of how well the newsletter met its objectives and **b** stated reasons for non-achievement of the objectives. **a** 10 objectives of the newsletter to foster evidence-based self-medication counseling are arranged in descending order according to the proportion of participants agreeing to the corresponding statements. Data on the objective ‘Promotion of motivation for discussing the current evidence base with colleagues’ were missing for one participant. **b** If participants stated reasons for disagreement, the answers (free-text) were subsequently assigned to four predefined categories by the investigators. Multiple categories could apply to each response (n [total] = 150)

All objectives of the professional newsletter were achieved for most of the participants (57–91%). More than 80% of them attributed positive changes in their knowledge (89%), skills (87–91%) and awareness (85%) to the professional newsletter. Between 67 and 77% of them attributed positive changes in their motivation to it. Practicability of reading in everyday working life was the least achieved objective with 57% of participants rating it positively (Fig. 3a). Achieving five out of the 10 objectives on the levels ‘skills’, ‘awareness’ and ‘motivation’ correlated significantly with an increasing number of newsletter issues read (Table 2).

**Table 2:** Correlation between achieving the objectives and number of read newsletter issues

Level	Objective	Correlation coefficient *Spearman’s rho*	*P* value
Knowledge	Support for obtaining an overview of the current evidence base	+ 0.111	0.175
Skills	Support for understanding scientific data	+ 0.126	0.125
	Support for appraising scientific data critically with regard to their clinical relevance	+ 0.222	0.006*
	Support for critically questioning information from pharmaceutical companies	+ 0.204	0.012*
	Support for counseling patients based on scientific evidence	+ 0.259	0.001*
Awareness	Raise of awareness of science-based counseling	+ 0.174	0.033*
Motivation	Promotion of motivation for searching scientific data as the basis for counseling	+ 0.072	0.379
	Promotion of motivation for reading scientific data as the basis for counseling	+ 0.162	0.047*
	Promotion of motivation for discussing the current evidence base with colleagues	+ 0.131	0.112
Practicability	Easy incorporation of reading in everyday working life	+ 0.025	0.762

*Statistically significant. Each statistically significant correlation coefficient represents a small relationship between achieving the objectives on a 6-point Likert scale and the number of newsletter issues read

### Barriers to the professional newsletter’s integration in everyday practice and suggestions for modifications to the concept

Table 3 presents the opinions of all participants about the preferred format of information presentation.

**Table 3:** Opinions about the future format of information presentation in the newsletter (n [total] = 150)

Aspect	Preferred format of information presentation	n	%
Comprehensiveness	Detailed description and explanation of the study methodology that was used and the associated results	21	14%
	Short presentation of study results with reference to the original literature for further information	92	61%
	Both apply equally	31	21%
	Do not know/not specified	6	4%
Recommendations	Neutral presentation of study data	33	22%
	Making recommendations	70	47%
	Both apply equally	41	27%
	Do not know/not specified	6	4%
Range of topics	Presentation of study data of several active substances regarding one field of indication as continuous series	65	43%
	Presentation of study data of single active substances regarding different fields of indication in turns	26	17%
	Both apply equally	54	36%
	Do now know/not specified	5	3%

The rounding of values may result in total amounts deviating from 100%

Participants who rated the objective ‘Easy incorporation of reading in everyday working life’ as not achieved, stated ‘newsletter-related reasons’ and ‘underlying conditions’ as reasons for non-achievement (Fig. 3b). “Lack of time” and “not enough staff in the pharmacy” were the most frequently mentioned ‘underlying conditions’. Therefore, and in general, some participants expressed their desire for more simplified and shorter newsletter issues with more emphasis on the results of the clinical trials instead of their methods. Other participants, however, were opposed to decreasing the complexity of the content. Instead, they preferred to read the professional newsletter in their leisure time*.* To increase the clarity and readability, some participants suggested that the information should be prepared more frequently in tables and figures. This way, implementation of the content into routine counseling might be improved*.* Doubts about the newsletter format per se were expressed occasionally, as was the wish for incorporation of the content into established software used in everyday practice (Table 4).

**Table 4:** Subscribers’ suggestions to improve the practicability of the professional newsletter

Subcategory of suggestions	Quotation example	Subscriber
Shortening	“The newsletter should be shorter. More emphasis should be placed on the results of the clinical trials [instead of the methods]”	Pharmacist, 4 years’ work experience
Additional short text version	“[I desire] a short summary and appraisal of trial results, if necessary, with reference to a more detailed version”	Pharmacist, 15 years’ work experience
Simplifying the content	“I would have to find time for [reading] during my daily work, unfortunately this is not the case with full capacity utilization in the pharmacy. It is difficult for me to read complex scientific contents ‘as a sideline’, [because] then I do not understand them. When I am interrupted again and again, unfortunately, nothing gets stuck in my head. But that is in the nature of things and not a criticism of the newsletter. I would not be in favor of making it ‘simpler’ and thus less scientific, just so that you can read it alongside your work. Then I would rather read it in my leisure time”	Pharmacist, 18 years’ work experience
Intensified graphical editing	“Although [it would be] elaborate, an intensified graphical editing of the results would be useful (postings, overviews). This would help establishing the information and findings for the entire [pharmacy] team faster and more effectively”	Pharmacist, 16 years’ work experience
	“[The content should be] clearer summarized, less continuous text, more bullet points, more illustrations/tables/diagrams (general overviews)”	Pharmacist, 1 year’s work experience
Incorporation in pharmacy software	“A newsletter is not the appropriate format [to foster evidence-based counseling]. The questions in practice are too manifold.[…] It would be better, if evidence-based information on OTC medicines could be incorporated in the pharmacy software”	Pharmacist, 8 years’ work experience

## Discussion

Providing self-medication counseling based on scientific evidence presents a substantial challenge for community pharmacists [6, 28]. So far, little has been known known about the development, implementation and utility of concepts for evidence-based counseling in community pharmacies. Previous concepts were mostly limited to a few self-medication indications and a small number of pharmacists. Furthermore, they did not focus on the need for appropriate information resources for routine counseling [29–32]. We developed a professional newsletter for all German community pharmacists that provided evidence-based information on common OTC medicines as well as instructions for searching and appraising scientific literature. The majority of participants in the evaluation survey rated the professional newsletter as helpful. In order to enhance a comprehensive application of such an information resource in daily routine, however, modifications to the concept, such as shortening the newsletter issues, and additional measures should be considered.

### Need for continuing information

Our survey evaluation revealed a small correlation between the subscribers’ perception of the support provided by the professional newsletter and the number of newsletter issues read. These findings suggest that pharmacists may benefit from more regular reading. Our results, however, might also be attributed to the characteristics of the participating subscribers themselves. It is possible that particularly motivated pharmacists with solid evidence-based skills read the professional newsletter more often and took part in the survey evaluation. Interestingly, pharmacists with relatively long-term professional experience participated in our survey. In other studies, more experienced pharmacists had a lower level of evidence-based knowledge and skills [7, 33]. To our knowledge, only a few studies have explored the effect of professional newsletters on healthcare professionals’ use of scientific evidence and therapeutic decisions [22, 23]. Results from those studies indicate that to foster EBP, long-term provision of a newsletter service is probably more useful than the provision of a huge number of newsletters in a short time.

### Ensuring the practicability of a professional newsletter

Despite that our professional newsletter was perceived as supportive for evidence-based self-medication counseling, the practicability of reading has to be improved. Therefore, shortening the newsletter issues should be considered. At the same time, it should be borne in mind that evidence-based counseling is more than just considering the results of clinical trials for decision-making. Before applying trial results in practice, clinical trial data have to be appraised for their internal and external validity [20]. In our professional newsletter, we consciously presented key aspects of methods, essential for the understanding of a clinical trial. This way, we attempted to make our appraisal of a trial’s validity transparent and comprehensible for the subscribers. Additionally, in the sense of EBP, community pharmacists are still responsible to evaluate the applicability of clinical trial data to their specific patient cases. As a consequence, it would be inappropriate to fulfill the desire of some of our survey participants not to present trial methods at all. In fact, subscribers should be provided at least with elementary methodical aspects from clinical trials (i.e. patient, intervention, control, outcome, and setting) [34]. However, summarizing the most relevant findings for everyday practice can be helpful.

Another possibility for increasing the practicability might be to make more recommendations, similar to clinical guidelines, an addition requested by almost half of the participants in our online survey. These results are in line with other studies. Pharmacists mostly favor using guidelines developed by renowned experts [8, 35]. However, making well-founded guideline recommendations would require many experts to come together, to read the relevant studies and to discuss their clinical relevance. This would take more time and thus entail higher financial costs [36, 37]. As a pragmatic concession, we included the element ‘Conclusion for practice’ in our professional newsletter, in which we summarized the main findings and consequences for counseling from our point of view.

### Necessity of additional measures

In relation to the total number of pharmacies in Germany (around 19,400 pharmacies with 52,000 pharmacists) [38], our professional newsletter reached only every tenth pharmacy (1975 subscribers). In order to foster a comprehensive implementation of EBP in German community pharmacies, too few pharmacists have subscribed to the newsletter, so far. The wide range of various newsletters pharmacists might receive in everyday practice could result in not appreciating the additional value of an EBP newsletter concept. Therefore, participation in continuing education sessions, public campaigns by professional societies, and further research projects should raise awareness about what EBP is and why it is needed to advise patients properly. If pharmacists recognize their need for acquiring evidence-based knowledge and skills, they might use corresponding educational resources, such as our professional newsletter, more extensively.

### Limitations of the evaluation survey

- We assessed the utility of the professional newsletter as perceived by the subscribers themselves. Therefore, an actual effect of the professional newsletter on the subscribers’ knowledge, skills and behavior as well as an improvement of EBP in the community pharmacy could not be shown.
- A validation of the survey was not performed, which may limit the accuracy of the results. However, it was pretested with four pharmacists to ensure completeness and feasibility.
- While all subscribers were invited several times to participate in the survey, only 9% of them completed the survey. This suggests that those who were motivated to read the professional newsletter were more likely to participate. As we informed the subscribers that the survey was conducted to adapt future newsletters to their needs, we also assume that critical newsletter subscribers were more likely to participate in the survey. Nevertheless, the low response rate requires caution with generalizations.
- The actual amount of time the subscribers spent on reading and viewing the website as well as the number of newsletter issues read could not be recorded. While all data in the evaluation survey were collected anonymously, social desirability bias may have resulted in an overestimation of the amount of reading.

## Conclusion

In order to implement EBP in their self-medication counseling, pharmacists need to continuously educate themselves. A nationwide provided professional newsletter can play a vital part in supporting pharmacists in evidence-based self-medication counseling. However, such an information resource needs to be better integrated in everyday working life. To ensure a high practicability for community pharmacists, the synthesized information should be presented concisely but should also meet the core principles of EBP, which include the critical appraisal of clinical trial data. Generally, information resources need to be accompanied by additional measures to close existing evidence-to-practice gaps. Future studies should further focus on the sustainable improvement of the professional newsletter concept and on fostering pharmacists’ awareness of EBP.

## Electronic supplementary material

Below is the link to the electronic supplementary material.

Supplementary file1 (PDF 144 kb)

Supplementary file2 (PDF 192 kb)

## Abbreviations

EBP: Evidence-based practice
OTC: Over-the-counter
