# Supplementary material for: Evidence-based self-medication: development and evaluation of a professional newsletter concept for community pharmacies
Source: Int J Clin Pharm. 2020 Jul 30;43(1):55–65. doi: 10.1007/s11096-020-01100-6 (PMC7878231; doi:10.1007/s11096-020-01100-6)
Supplement: Supplementary file 1 — Supplementary file1 (PDF 144 kb) [file 11096_2020_1100_MOESM1_ESM.pdf]

Moritz K*, Seiberth JM*, Schiek S, Bertsche T (* equal contributing)
Dept. of Clinical Pharmacy, Institute of Pharmacy, Faculty of Medicine, Leipzig University and Drug Safety Center, University Hospital Leipzig and Leipzig University Leipzig, Germany; email to thilo.bertsche@uni-leipzig.de

Online Resource 1

Topics of the published newsletter issues up to and including July 2018 (original language: German)

Issue number	Publishing date	Page count†	Included section	Section title
1	May 23, 2017	6.0	Good to know	Oxford league table of analgesics in acute pain
			Background	Efficacy tests of analgesics for acute pain on the basis of single doses
			Infobox	What is a systematic review and a Cochrane review?
			Infobox	Number needed to treat (NNT)
2	June 13, 2017	9.3	Focus	Efficacy of per oral analgesics in self-medication for acute pain
			Background	Background of clinical trials for acute pain
			Infobox	Success rate in pain studies
			Infobox	Study concepts frequently used in acute pain
3	June 27, 2017	4.5	Focus	Cochrane review: Acetaminophen for non-specific low back pain
			Background	Clinical guideline recommendations on acetaminophen for low back pain
			Infobox	The GRADE approach and its connection to clinical guidelines
4	July 11, 2017	8.6	Good to know	Combination of analgesics for non-specific low back pain
			Good to know	Acetaminophen for low back pain during pregnancy
			Infobox	Pain measurement scales
			Infobox	Roland Morris Disability Questionnaire (RMDQ) for the measurement of disability caused by back pain
			Infobox	Quebec Back Pain Disability Scale (QBPDS) for the measurement of disability caused by back pain
			Infobox	Fingertip-to-floor test for mobility measurement of the backbone
5	July 25, 2017	7.9	Good to know	Headache classifications
			Good to know	Exclusions for self-treatment of headache: When should the pharmacist refer patients to a physician?
			Infobox	AMSTAR (A MeaSurement Tool to Assess Systematic Reviews)
6	August 8, 2017	3.7	Infobox	Clinical guidelines
7	August 22, 2017	11.7	Focus	Acetylsalicylic acid for episodic tension type headache
			Background	Clinical guideline recommendations on acetylsalicylic acid for tension type headache
8	September 12, 2017	8.8	Focus	Non-steroidal anti-inflammatory drugs for non-specific low back pain
			Background	Clinical guideline recommendations on non-steroidal anti-inflammatory drugs for low back pain
			Infobox	Quality assurance of scientific publications: The peer-review
9	September 26, 2017	9.6	Focus	Efficacy and safety of single doses of decongestants for the common cold

Moritz K*, Seiberth JM*, Schiek S, Bertsche T (* equal contributing)
Dept. of Clinical Pharmacy, Institute of Pharmacy, Faculty of Medicine, Leipzig University and Drug Safety Center, University Hospital Leipzig and Leipzig University Leipzig, Germany; email to thilo.bertsche@uni-leipzig.de

Issue number	Publishing date	Page count†	Included section	Section title
			Infobox	Mean difference (MD) and standardized mean difference (SMD) as effect measures for continuous outcomes
10	October 10, 2017	9.7	Good to know<br>Infobox	Guideline on conducting clinical trials for tension type headache<br>Headache diary
11	October 24, 2017	8.6	Focus<br>Background	Ibuprofen for episodic tension type headache<br>Clinical guideline recommendations on ibuprofen for tension type headache
12	November 14, 2017	12.2	Focus<br>Background<br>Infobox	Efficacy and safety of multiple doses of decongestants for the common cold<br>Clinical guideline recommendations on decongestants for the common cold<br>Odds Ratio (OR) as an effect measure for dichotomous outcomes
13	November 28, 2017	7.8	Focus<br>Infobox	Comfrey root for non-specific low back pain<br>Oswestry Disability Index (ODI) for measurement of disability caused by back pain
14	December 12, 2017	9.2	Background<br>Background<br>Infobox	Characterization of the comfrey preparations proved in clinical trials<br>Monographies and clinical guideline recommendations on comfrey root for non-specific low back pain<br>Monographs from the Committee on Herbal Medicinal Products (HMPC): Evidence for herbal medicinal products
15	January 9, 2018	12.9	Short profile<br>Focus<br>Background<br>Update	Antihistamines for the common cold<br>Monotherapy with antihistamines for the common cold<br>Available antihistamines and clinical guideline recommendations for the common cold<br>Decongestants for the common cold
16	February 13, 2018	14.6	Short profile<br>Background<br>Focus	Combination of ibuprofen and caffeine for acute pain<br>Available products with analgesics and caffeine and clinical guideline recommendations<br>Cochrane-review: Combination of ibuprofen and caffeine for acute pain
17	March 13, 2018	12.3	Spotlight<br>Infobox	Combination of ibuprofen and caffeine for acute pain: Clinical trial with 400 mg ibuprofen and 100 mg caffeine<br>Levels of evidence and grades of recommendations used by the Oxford Centre for Evidence-based Medicine
18	April 18, 2018	12.7	Short profile<br>Background<br>Spotlight	Nose spray with carrageenan for the common cold<br>Available sprays with glycerin/trypsin, carrageenan and hydroxy propyl methyl cellulose for the common cold<br>Evidence base of carrageenan nose spray for the common cold
19	May 8, 2018	15.0	Short profile	Mannose for prophylaxis of recurrent urinary tract infections

Moritz K*, Seiberth JM*, Schiek S, Bertsche T (* equal contributing)
Dept. of Clinical Pharmacy, Institute of Pharmacy, Faculty of Medicine, Leipzig University and Drug Safety Center, University Hospital Leipzig and Leipzig University Leipzig, Germany; email to thilo.bertsche@uni-leipzig.de

Issue number	Publishing date	Page count†	Included section	Section title
			Background	Recurrent urinary tract infections, available products with mannose and clinical guideline recommendations
			Spotlight	Evidence base of mannose for prophylaxis of recurrent urinary tract infections
20	June 12, 2018	9.6	Short profile	Mouth spray with glycerin and trypsin for the common cold
			Spotlight	Evidence base of glycerin and trypsin-based mouth spray for the common cold
			Short profile	Nose spray with hydroxy propyl methyl cellulose for the common cold
			Spotlight	Evidence base of hydroxy propyl methyl cellulose nose spray for the common cold
21	July 10, 2018	17.3	Short profile	Minoxidil for female pattern hair loss
			Background	Androgenetic alopecia, available products with minoxidil and clinical guideline recommendations
			Focus	Evidence base of minoxidil for female pattern hair loss

† 300 words corresponds to one page (references, glossary and legal notice not considered)
