# Supplementary material for: Evidence-based self-medication: development and evaluation of a professional newsletter concept for community pharmacies
Source: Int J Clin Pharm. 2020 Jul 30;43(1):55–65. doi: 10.1007/s11096-020-01100-6 (PMC7878231; doi:10.1007/s11096-020-01100-6)
Supplement: Supplementary file 2 — Supplementary file2 (PDF 192 kb) [file 11096_2020_1100_MOESM2_ESM.pdf]

Moritz K*, Seiberth JM*, Schiek S, Bertsche T (* equal contributing)
Dept. of Clinical Pharmacy, Institute of Pharmacy, Faculty of Medicine, Leipzig University and Drug Safety Center, University Hospital Leipzig and Leipzig University, Leipzig, Germany; email to thilo.bertsche@uni-leipzig.de

Online Resource 2

Translated questions from the original German survey.

1. Up to and including March 2018 seventeen issues of EVInews were published. How many issues have you read since you received the newsletter?

- ☐ More than 15 issues
- ☐ 11 to 15 issues
- ☐ 6 to 10 issues
- ☐ 1 to 5 issues
- ☐ No issue

Questions 2 and 3 were asked only of survey participants who had read at least one newsletter issue.

2. When do you read EVInews?

- ☐ I read the newsletter during my working hours.
- ☐ I read the newsletter in my leisure time.
- ☐ Both apply equally.

3. How do you read EVInews?

- ☐ I skim through the newsletter.
- ☐ I work through the newsletter.
- ☐ Both apply equally.

4. How do you use EVInews for your counseling practice?
Multiple choice possible

- ☐ We exchange our views about the newsletter in the pharmacy team.
- ☐ I/we use the newsletter to compile counseling recommendations.
- ☐ I use the newsletter as a basis for further searches.
- ☐ I use the newsletter as a general work of reference.
- ☐ Other: ____ (free-text)

Moritz K*, Seiberth JM*, Schiek S, Bertsche T (* equal contributing)
Dept. of Clinical Pharmacy, Institute of Pharmacy, Faculty of Medicine, Leipzig University and Drug Safety Center, University Hospital Leipzig and Leipzig University, Leipzig, Germany; email to thilo.bertsche@uni-leipzig.de

5. EVInews contains information about the present scientific state of knowledge. The newsletter provides the information in different sections occurring with varying frequency.
In your opinion, how useful is the information presentation in the newsletter generally?

	entirely useful	mainly useful	rather useful	rather useless	mainly useless	entirely useless	do not know
**Section 'Focus':** Presentation of study findings with the best available scientific evidence on an active substance or a substance group for a field of indication	☐	☐	☐	☐	☐	☐	☐
**Section 'Background':** Presentation of background information (e.g. clinical guidelines, status of approval) to integrate study findings into the overall context	☐	☐	☐	☐	☐	☐	☐
**Section 'Good to know':** Presentation of study findings with additional information (e.g. special patient groups like children and pregnant women)	☐	☐	☐	☐	☐	☐	☐
**Section 'Spotlight':** Presentation of topics, which are currently discussed in the specialized press (e.g. new available active substances)	☐	☐	☐	☐	☐	☐	☐
**Section 'Update':** Presentation of new study findings on topics already covered in previous newsletters	☐	☐	☐	☐	☐	☐	☐
**Section 'Short profile':** Tabular summary of the most relevant findings for counseling, which are discussed in the other sections in detail.	☐	☐	☐	☐	☐	☐	☐
**Section 'Questions to the physician':** Recommendations of practicing physicians or professional societies to support the incorporation of medical expertise into counseling practice	☐	☐	☐	☐	☐	☐	☐
**Section 'Infobox (Search)':** Tips for the literature search in the community pharmacy	☐	☐	☐	☐	☐	☐	☐
**Section 'Infobox (Technical terms)':** Explanation of selected technical terms and methods for the critical appraisal of clinical trial data	☐	☐	☐	☐	☐	☐	☐
**'Conclusion for practice':** Summary of the main findings at the end of each section	☐	☐	☐	☐	☐	☐	☐
**'Glossary':** Short definition of used technical terms at the end of the newsletter	☐	☐	☐	☐	☐	☐	☐

6. We want to design the content of EVInews in the way that you can use the newsletter optimally for your counseling practice in the future.

a) In your opinion, what format of information presentation is more useful for your counseling practice?

☐ Detailed description and explanation of the study methodology that was used and the associated results.
☐ Short presentation of study results with reference to the original literature for further information.
☐ Both apply equally.

☐ Do not know.

Moritz K*, Seiberth JM*, Schiek S, Bertsche T (* equal contributing)
Dept. of Clinical Pharmacy, Institute of Pharmacy, Faculty of Medicine, Leipzig University and Drug Safety Center, University Hospital Leipzig and Leipzig University, Leipzig, Germany; email to thilo.bertsche@uni-leipzig.de

b) In your opinion, what format of information presentation is more useful for your counseling practice?

- ☐ Neutral presentation of study data.
- ☐ Making recommendations.
- ☐ Both apply equally.

- ☐ Do not know.

c) In your opinion, what format of information presentation is more useful for your counseling practice?

- ☐ Presentation of study data of several active substances regarding one field of indication as continuous series.
- ☐ Presentation of study data of single active substances regarding different fields of indication in turns.
- ☐ Both apply equally.

- ☐ Do not know.

7. How much do you agree with the following statements?

	entirely agree	mainly agree	rather agree	rather disagree	mainly disagree	entirely disagree
Reading the newsletter can be easily incorporated into everyday working life.	☐	☐	☐	☐	☐	☐
The newsletter helps me to obtain an overview of the current evidence base.	☐	☐	☐	☐	☐	☐
The newsletter has raised my awareness of science-based counseling.	☐	☐	☐	☐	☐	☐
The newsletter helps me to critically question information from pharmaceutical companies.	☐	☐	☐	☐	☐	☐
The newsletter motivates us to discuss the current evidence base with colleagues.	☐	☐	☐	☐	☐	☐
The newsletter helps me to counsel my patients based on scientific evidence.	☐	☐	☐	☐	☐	☐
The newsletter motivates me to <u>search</u> for scientific data* as the basis for my counseling practice.	☐	☐	☐	☐	☐	☐
The newsletter motivates me to <u>read</u> scientific data* as the basis for my counseling practice.	☐	☐	☐	☐	☐	☐
The newsletter helps me to <u>understand</u> scientific data*.	☐	☐	☐	☐	☐	☐
The newsletter helps me to <u>appraise</u> scientific data* critically with regard to their clinical relevance.	☐	☐	☐	☐	☐	☐

*** Please note**: The term ‘scientific data’ refers to clinical trials, systematic reviews and clinical guidelines in this survey.

Questions 8 to 17 were asked only of survey participants who had (rather/mainly/entirely) disagreed with the corresponding previous statement.

8. You (rather/mainly/entirely) <u>disagreed</u> with the statement: *‘Reading the newsletter can be easily incorporated into everyday working life.’*
What does need to change so that you agree with that statement?

Free-text

Moritz K*, Seiberth JM*, Schiek S, Bertsche T (* equal contributing)
Dept. of Clinical Pharmacy, Institute of Pharmacy, Faculty of Medicine, Leipzig University and Drug Safety Center, University Hospital Leipzig and Leipzig University, Leipzig, Germany; email to thilo.bertsche@uni-leipzig.de

9. **You (rather/mainly/entirely) disagreed with the statement:** *'The newsletter helps me to obtain an overview of the current evidence base.'*
   What does need to change so that you agree with that statement?

   Free-text

10. **You (rather/mainly/entirely) disagreed with the statement:** '*The newsletter has raised my awareness of science-based counseling.'*
    What does need to change so that you agree with that statement?

    Free-text

11. **You (rather/mainly/entirely) disagreed with the statement:** *'The newsletter helps me to critically question information from pharmaceutical companies.'*
    What does need to change so that you agree with that statement?

    Free-text

12. **You (rather/mainly/entirely) disagreed with the statement:** *'The newsletter motivates us to discuss the current evidence base with colleagues.'*
    What does need to change so that you agree with that statement?

    Free-text

13. **You (rather/mainly/entirely) disagreed with the statement:** *'The newsletter helps me to counsel my patients based on scientific evidence.'*
    What does need to change so that you agree with that statement?

    Free-text

14. **You (rather/mainly/entirely) disagreed with the statement:** *'The newsletter motivates me to **search** for scientific data* as the basis for my counseling practice.'*
    What does need to change so that you agree with that statement?

    Free-text

*** Please note**: The term 'scientific data' refers to clinical trials, systematic reviews and clinical guidelines in this survey.

15. **You (rather/mainly/entirely) disagreed with the statement:** *'The newsletter motivates me to **read** scientific data* as the basis for my counseling practice.'*
    What does need to change so that you agree with that statement?

    Free-text

*** Please note**: The term 'scientific data' refers to clinical trials, systematic reviews and clinical guidelines in this survey.

16. **You (rather/mainly/entirely) disagreed with the statement:** *'The newsletter helps me to **understand** scientific data*.'*
    What does need to change so that you agree with that statement?

    Free-text

*** Please note**: The term 'scientific data' refers to clinical trials, systematic reviews and clinical guidelines in this survey.

Moritz K*, Seiberth JM*, Schiek S, Bertsche T (* equal contributing)
Dept. of Clinical Pharmacy, Institute of Pharmacy, Faculty of Medicine, Leipzig University and Drug Safety Center, University Hospital Leipzig and Leipzig University, Leipzig, Germany; email to thilo.bertsche@uni-leipzig.de

17. You (rather/mainly/entirely) disagreed with the statement: *'The newsletter helps me to **appraise** scientific data* critically with regard to their clinical relevance.'*
What does need to change so that you agree with that statement?

Free-text

*** Please note**: The term 'scientific data' refers to clinical trials, systematic reviews and clinical guidelines in this survey.

18. Approximately when did you first subscribe to EVInews?

Drop down list with month and year

19. How old are you?

____ (free-text) years

20. Please indicate your gender.

- ☐ Male
- ☐ Female

21. What is your profession?

- ☐ Pharmacist
- ☐ Pharmaceutical engineer
- ☐ Pharmacist's assistant
- ☐ Pharmaceutical technical assistant
- ☐ Pharmaceutical assistant
- ☐ Pre-approbation trainee pharmacist
- ☐ Pharmaceutical technical assistant trainee
- ☐ Other: ____ (free-text)

Question 22 was asked only of survey participants who had indicated being a pharmacist.

22. Do you own a community pharmacy?

- ☐ Yes
- ☐ No

23. Where do you work?
Please indicate all sectors you presently work.

- ☐ Community pharmacy
- ☐ Hospital pharmacy
- ☐ German Armed Forces Pharmacy
- ☐ Pharmaceutical company
- ☐ Administration
- ☐ Medical insurance company
- ☐ Academics
- ☐ Publishing company
- ☐ Other: ____ (free-text)

24. Please indicate your work experience in the community pharmacy.

____ (free-text) years

Question 25 was asked only of survey participants who had indicated working in a community pharmacy.

Moritz K*, Seiberth JM*, Schiek S, Bertsche T (* equal contributing)
Dept. of Clinical Pharmacy, Institute of Pharmacy, Faculty of Medicine, Leipzig University and Drug Safety Center, University Hospital Leipzig and Leipzig University, Leipzig, Germany; email to thilo.bertsche@uni-leipzig.de

25. Taking into account your weekly working time in the community pharmacy: How frequently do you work in counter sales?

- ☐ Always
- ☐ Frequently
- ☐ Sometimes
- ☐ Seldom
- ☐ Never
